# Non-pharmacological Approaches to Apathy and Depression: A Scoping Review of Mild Cognitive Impairment and Dementia

**DOI:** 10.3389/fpsyg.2022.815913

**Published:** 2022-02-16

**Authors:** Hikaru Oba, Ryota Kobayashi, Shinobu Kawakatsu, Kyoko Suzuki, Koichi Otani, Kazushige Ihara

**Affiliations:** ^1^Graduate School of Health Sciences, Hirosaki University, Hirosaki, Japan; ^2^Department of Psychiatry, Yamagata University Faculty of Medicine, Yamagata, Japan; ^3^Department of Neuropsychiatry, Aizu Medical Center, Fukushima Medical University, Fukushima, Japan; ^4^Department of Behavioral Neurology and Cognitive Neuroscience, Tohoku University Graduate School of Medicine, Sendai, Japan; ^5^Department of Social Medicine, Hirosaki University Graduate School of Medicine, Hirosaki, Japan

**Keywords:** Alzheimer’s disease, cognitive impairment, behavioral and psychological symptoms of dementia, intervention, randomized controlled trial, treatment

## Abstract

Apathy and depression are frequently observed as behavioral and psychological symptoms of dementia, respectively, and are important for ensuring adequate care. This study aims to explore effective non-pharmacological interventions for apathy and depression with mild cognitive impairment (MCI) and dementia. Five search engines including PubMed, Scopus, CINAHL, PsycInfo, and Web of Science were used to extract relevant studies. Inclusion criteria were studies that involved participants who were diagnosed with MCI or dementia, included quantitative assessments of each symptom, and employed randomized controlled trials. Twenty studies were extracted, with interventions have been conducted in care facilities, the community, and hospitals. Participants in many studies had MCI or mild-to-moderate dementia but were not diagnosed with the subtypes of dementia. Few studies had set apathy and depression as the primary outcomes of non-pharmacological interventions. The findings suggested that emotional and stimulation-oriented approaches to apathy and depression would be useful for people with MCI or mild-to-moderate dementia. It would be helpful for therapists to assess the clinical features of the target symptoms for selecting suitable interventions. Additionally, increasing the number of randomized controlled trials focusing on apathy or depression as primary outcomes would offer a more definite conclusion for future systematic reviews.

## Introduction

Apathy and depression are frequently observed in people with dementia. For instance, 48% of people with moderate dementia exhibited apathy and 33.5% of them also had depression (Robert et al., 2009). While the most frequent psychiatric symptom in long-term care facilities is agitation/aggression (33.7%), apathy (16.0%) is the third most frequent symptom (Arai et al., 2017). Studies have also reported that 10–20% of older people with dementia living in the community have apathy or depression, both of which are independently associated with cognitive decline (Vaingankar et al., 2017; Van Dalen et al., 2018). A recent systematic review and meta-analysis revealed that the prevalence rate of apathy and depression in dementia was 54 and 39%, respectively (Leung et al., 2021). Among the behavioral and psychological symptoms of dementia (BPSD), caregivers do not pay adequate attention to apathy and depression compared to agitation/aggression; thus, apathy or depression in people with dementia is often ignored. Moreover, the care staff in long-term care facilities who are aware of apathy and depression are often underconfident about dealing with these symptoms (Oba et al., 2020).

Apathy is defined as a loss of motivation (Marin, 1991; Starkstein and Leentjens, 2008). The diagnostic criteria of apathy in neuropsychiatric diseases comprise three aspects: loss of goal-directed behavior, loss of goal-directed cognition, and loss of goal-directed emotion (Starkstein et al., 2005; Robert et al., 2009). These aspects are further divided into loss of spontaneous or environment-stimulated responses (Robert et al., 2009). Other criteria have emphasized the behavioral aspects of apathy, except for the psychological interpretation, which defines it as the quantitative reduction in self-generated voluntary and purposeful behaviors (Levy and Dubois, 2006). Depression is characterized by a depressive mood and loss of interest or pleasure in the Diagnostic and Statistical Manual of Mental Disorders-fifth edition (American Psychiatric Association, 2013). The diagnostic criteria of apathy and depression reveal that these symptoms are similar to each other, particularly in terms of loss of motivation or interest. However, apathy and depression should be clearly distinguished; although these symptoms occasionally coexist, apathy can independently occur even if patients do not have depression (Levy and Dubois, 2006).

Non-pharmacological intervention is the first choice to treat BPSD because older adults tend to experience adverse effects of medicines (Lenzer, 2005; International Psychogeriatric Association, 2012). American Psychiatric Association (2007), Work Group on Alzheimer’s Disease and Other Dementias, proposed four approaches to non-pharmacological interventions for BPSD: (1) behavior-oriented approaches; (2) emotion-oriented approaches including reminiscence therapy, validation therapy, and supportive psychotherapy; (3) cognition-oriented approaches including reality orientation and skills training; and (4) stimulation-oriented approaches including recreational activities, art therapies, exercise, and music therapies. These approaches are not completely independent, with some overlapping others. Although this framework is useful for understanding the types of non-pharmacological approaches, previous studies have suggested the effect of other factors, including personalization in tailored activities and person-centered care for BPSD (Chenoweth et al., 2009; Lu et al., 2021).

Cohen-Mansfield (2007) has pointed out that treatment plans, which include a goal, needs, and preferences, dictate the selection of the non-pharmacological intervention for BPSD. Evidence regarding non-pharmacological interventions for dementia is inconsistent among various studies; one of the reasons could be that specific psychiatric symptoms, such as apathy and depression, are not the primary goal of these studies. It is thus important to develop targeted non-pharmacological interventions for depression and apathy because these symptoms cause physical and psychological decline in people with dementia. This review aims to explore effective non-pharmacological interventions for apathy and depression with dementia.

## Materials and Methods

The review process was conducted in accordance with the Preferred Reporting Items for Systematic Reviews and Meta-Analyses for Scoping Reviews guidelines (Tricco et al., 2018).

### Inclusion Criteria

The inclusion criteria were as follows: (1) participants had to be diagnosed with dementia or mild cognitive impairment (MCI), (2) quantitative assessment of apathy or depression using any scale had to be conducted, and (3) the study had to be a randomized controlled trial.

### Search Strategy, Study Selection, and Evaluation Criteria

The search was conducted on September 25, 2020, using five search engines: PubMed, Scopus, CINAHL, PsycInfo, and Web of Science. The following keywords were used as: (“dementia” OR “Alzheimer” OR “cognitive impairment”) AND (“depression” OR “depressive symptom” OR “apathy” OR “abulia” OR “amotivation” OR “passivity”) AND “non-pharmacological” AND (“training” OR “rehabilitation” OR “treatment” OR “therapy” OR “intervention” OR “trial” OR “management”). Two researchers read all the abstracts of the articles that were generated to confirm that they targeted apathy or depression, and then read the entire texts of the articles that were finally extracted. The researchers held discussions when they disagreed on their respective judgments of the criteria.

The definition of non-pharmacological approaches was set based on the framework of the American Psychiatric Association (2007), Work Group on Alzheimer’s Disease and Other Dementias. This framework prepares four categories for non-pharmacological interventions: behavior-oriented, emotion-oriented, cognitive-oriented, and stimulation-oriented approaches, as mentioned above. We judged which category best described the intervention of each study we reviewed, although some studies were judged as belonging to more than one category. For example, cognitive stimulation therapy was classified into both cognition-orientated and stimulation-orientated approaches. We confirmed whether the term “cognitive stimulation” was used only to activate cognitive function. If the intervention focused only on cognitive training or rehabilitation, it was classified as belonging to the cognition-oriented approach. Moreover, we classified interventions not belonging to any of the four mentioned categories as the “other approach,” such as mindfulness-based intervention and psychoeducation to participants.

The severity of dementia for each study was judged by the description in the text or the score of the cognitive test. For studies using the Mini-Mental State Examination (MMSE), the mean scores of the intervention group ranging from 24 to 27 were defined as MCI, 20 to 23 as mild dementia, 15 to 19 as moderate dementia, and <15 as severe dementia. For studies using the Montreal Cognitive Assessment (MoCA), the mean scores of the intervention group ranging from 21 to 25 were defined as MCI, and scores <21 were defined as mild-to-moderate dementia (Lai et al., 2020). Additionally, for studies using the Clinical Dementia Rating (CDR), the mean rank of the intervention group was considered with CDR 0.5 being defined as MCI, CDR 1 as mild dementia, CDR 2 as moderate dementia, and CDR 3 as severe dementia.

The effect of the intervention of each study was evaluated based on the following criteria: (1) the quantitative scores based on the scales, (2) the intervention group (IG) results if the control group (CG) conducted continuous daily care, and (3) for both IG and CG, when the latter received a different kind of intervention than that of the IG. The intervention was judged to be “effective” when the score of the apathy or depression scale in the IG did not significantly change but that of the CG was significantly worse, and if the intervention succeeded at any stage of the evaluation.

## Results

### Search Process

**Figure 1:**
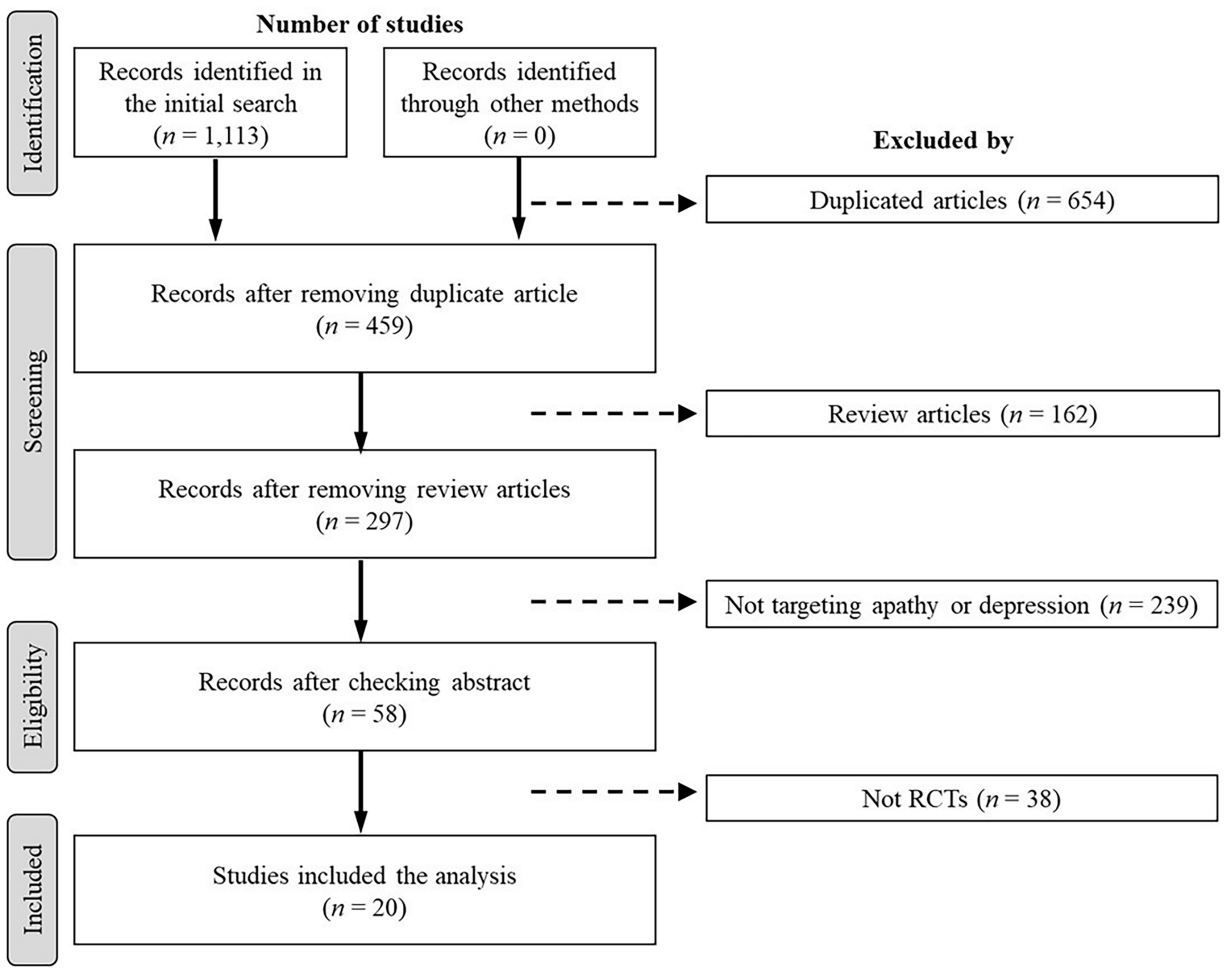
Study flow chart.

A total of 1,113 studies were identified in the initial search. A flowchart of the search process is depicted in Figure 1. Duplicate (*n* = 654) and review (*n* = 162) studies were first removed in the screening process; then, studies that did not target apathy or depression (*n* = 239) were excluded. The entire texts of 58 studies were read by two researchers and, finally, 20 studies that fulfilled the inclusion criteria were selected. The characteristics of each study are shown in Table 1.

**Table 1:** Randomized controlled trial of non-pharmacological interventions for apathy and depression.

Authors	*n*	Recruitment	Participants	Dementia severity	Measures	Depression score at baseline Mean (*SD*)	Apathy score at baseline Mean (*SD*)	Interventions	Primary outcome	Main results	Evaluation
Alves et al. (2014)	IG: *n* = 10 CG: *n* = 7	Day care and long-term older adult center	Patients with MCI and mild-to-moderate dementia	MCI and mild to moderate	Cognitive function: MMSE Depression: GDS (version unclear) Apathy: Not measured	IG: 11.3 (5.33) CG: 9 (4.87)		IG: Standard intervention group of CST (CA, SA) CG: Waiting list (No intervention except for the usual care)	Cognitive function, IADL, and therapy experience	Changed score of pre-post intervention was not significant between both groups (*p* = 0.84).	Ineffective
Bergamaschi et al. (2013)	IG: *n* = 16 CG: *n* = 16	Hospital	Patients with AD who have taken donepezil.	Mild to moderate	Cognitive function: MMSE Depression: CSDD Apathy: Not measured	IG: 29.37 (4.47) CG: 27.19 (8.28)		IG: Cognitive training (CA) CG: Multiple sessions of non-specific cognitive activities (SA)	Not clearly described	CSDD score was not significant between both groups at pre-post evaluation (*p* = 0.84).	Ineffective
Brooker et al. (2011)	IG: *n* = 144 CG: *n* = 149	Extra care housing	Residents with dementia	Moderate	Cognitive function: MMSE Depression: GDS-15 Apathy: Not measured	IG: 6.3 (SE = 0.5) CG: 5.3 (SE = 0.4)		IG: Enriched opportunities program (OA) CG: Project support worker coach (OA)	Not clearly described	IG group showed significant improvement of GDS score at 6 (*p* < 0.001), 12 (*p* < 0.001), and 18 months (*p* < 0.001).	Effective
Fernández-Calvo et al. (2015)	IG: *n* = 25 CG: *n* = 30	The Alzheimer’s Association of Salamanca	Patients with AD	Mild	Cognitive function: MMSE Depression: CSDD Apathy: Not measured	IG: 8.32 (2.14) CG: 7.83 (1.98)		IG: Multi-intervention Program (CA, SA) CG: Waiting list	Unawareness	CSDD in IG group scored lower than CG at post-treatment assessment (*p* < 0.05), *d* = 0.23, CI (−0.30, 0.76).	Effective
Gomez-Soria et al. (2020)	IG: *n* = 54 CG: *n* = 68	Hospital	People with MCI	MCI	Cognitive function: MMSE Depression: GDS-15 Apathy: Not measured	IG: 2.93 (2.60) CG: 3.14 (2.89)		IG: Cognitive stimulation (CA) CG: No intervention	Cognitive function	GDS score did not show a significant difference between both groups at post-test (*p* = 0.600) and 6 months post-test (*p* = 0.600).	Ineffective
Hattori et al. (2011)	IG: *n* = 20 CG: *n* = 19	Outpatient clinic at hospital	Patients with AD	Mild	Cognitive function: MMSE Depression: GDS-30 Apathy: SAS	IG: 4.3 (2.8) CG: 2.3 (1.8)	IG: 15.9 (7.1) CG: 13.0 (4.7)	IG: Art therapy (SA) CG: Calculation drill (CA)	Not clearly described	GDS score was not improved in both groups (IG, *p* = 0.294; CG, *p* = 0.466) at post-test. SAS score was improved only in IG (*p* = 0.014) at post-test (CG, *p* = 0.090).	Effective (Apathy) Ineffective (Depression)
Hsieh et al. (2010)	IG: *n* = 29 CG: *n* = 32	Nursing home	Residents with dementia	Mild	Cognitive function: CDR Depression: GDS-15, NPI Apathy: AES, NPI	GDS IG: 7.79 (1.83) CG: 7.41 (1.76) NPI IG: 2.83 (4.06) CG: 1.97 (3.83)	AES-C (Behavior, Emotion, Cognition) IG: 9.55 (1.57), 4.59 (1.05), 17.79 (2.38) CG: 8.94 (2.50), 4.00 (0.88), 16.19 (3.40) NPI IG: 3.28 (3.89) CG: 2.25 (3.07)	IG: Reminiscence Group Therapy (EA) CG: not described	Depressive symptoms and apathy	GDS and NPI depression subscale scores were significantly improved in IG (GDS, *p* = 0.003; NPI, *p* = 0.028). AES behavior subscale score was improved in IG (*p* = 0.002), but other subscale scores of AES and NPI apathy subscale score were not improved.	Effective (Apathy) Effective (Depression)
Huang et al. (2019).	IG: *n* = 36 CG: *n* = 38	Hospital and long-term care facilities	People with dementia	Mild	Cognitive function: MMSE Depression: GDS (version unclear) Apathy: Not measured	IG: 4.83 (2.57) CG: 4.95 (1.93)		IG: Tai Chi exercise (SA) CG: Only routine treatments and personalized daily care (OA)	Not clearly described	GDS score in IG was significantly improved between baseline and 10 months (*p* < 0.05, *d* = 0.35), and GDS score in IG was lower than that in CG at 10 months (*p* < 0.05, *d* = 0.87)	Effective
Lai et al. (2020)	IG: *n* = 50 CG: *n* = 50	Community	Older adults with dementia and caregivers	Mild to moderate	Cognitive function: MoCA Depression: RMBPC Apathy: Not measured	IG: 20.877 (5.56) CG: 21.07 (5.64)		IG: Dementia care education activity scheduling (OA) CG: Usual dementia care education (OA)	Caring role	The between and within-group differences were significant between both groups (between, *p* < 0.05, *d* = 0.47; within, *p* < 0.05, *d* = 0.50). RMBPC score in IG was significantly improved.	Effective
Larouche et al. (2019)	IG: *n* = 23 CG: *n* = 22	Community	Older adults with amnestic MCI	MCI	Cognitive function: MoCA Depression: GDS-30 Apathy: Not measured	IG:8.2 (SE 1.2) CG: 7.7 (SE 1.2)		IG: Mindfulness-based intervention (OA) CG: Psychoeducation-based intervention (OA)	Depression	GDS score of both interventions was significantly improved (time effect, *p* = 0.033; condition effect, *p* = 0.652; interaction between time and condition, *p* = 0.864).	Effective
Lin et al. (2019).	IG: *n* = 43 CG: *n* = 48	Long-term care facilities	Residents with dementia	Moderate	Cognitive function: MMSE Depression: CSDD Apathy: Not measured	IG: 3.79 (2.57) CG: 5.04 (4.03)		IG: Creative expression therapy (SA) CG: Standard cognitive training (CA)	Not clearly described	CSDD score of IG was significantly improved at post-test (*p* = 0.001, *d* = 1.54), which was maintained at follow-up (*p* = 0.012, *d* = 0.93).	Effective
Olsen et al. (2016)	IG: *n* = 23 CG: *n* = 25	Nursing home	Residents with dementia or cognitive deficit	Moderate to severe	Cognitive function: MMSE Depression: CSDD Apathy: Not measured	IG: 8.35 (4.65) CG: 6.88 (4.70)		IG: Animal-assisted activities (SA) CG: Usual care	Depression, agitation, and QOL	CSDD score of IG was not significantly different at pre-post (T1-T0) assessment (*p* = 0.171), but significantly improved between baseline and follow-up (T2-T0, *p* = 0.037).	Effective
Pérez-Ros et al. (2019)	IG: *n* = 47 CG: *n* = 72	Nursing home	Residents with dementia	Moderate	Cognitive function: MMSE Depression: GDS-15, CSDD Apathy: Not measured	GDS IG: 8.31 (5.78) CG: 9.77 (6.98) CSDD IG: 5.00 (4.53) CG: 8.03 (5.89)		IG: Preference for listening to music (SA) CG: Occupational therapy programs with no music-based intervention (SA)	Functional, cognitive, and emotional dimensions	GDS score in IG was maintained, but, in CG, it was worsened at post-test (*p* < 0.01). CSDD score was not different in both groups at post-test.	Effective
Pongan et al. (2017).	IG: *n* = 31 CG: *n* = 28	Memory clinic	Patients with AD	Mild	Cognitive function: MMSE Depression: GDS Apathy: Not measured	IG: 8.81 (SE 5.99) CG: 8.79 (SE 6.23)		IG: Singing intervention (SA) CG: Painting intervention (SA)	Pain	GDS score of CG (painting intervention) was significantly improved (interaction time*group: *p* = 0.01).	Effective
Reverté-Villarroya et al. (2020)	IG: *n* = 13 CG: *n* = 15	Hospital	Patients with dementia and their caregivers	Moderate	Cognitive function: MMSE Depression: GDS-30, NPI Apathy: NPI	IG: 4.85 (0.68) CG: 5.07 (0.88)	Not described	IG: Routine clinical practice and educational nursing education for the family caregivers (OA) CG: Routine clinical practice	BPSD	GDS score of IG was worsened at post-test (*p* < 0.001). Although NPI was used to assess apathy, quantitative assessment was not conducted.	Ineffective
Schmitter-Edgecombe and Dyck (2014)	IG: *n* = 23 CG: *n* = 23	Community	Care-dyads	MCI and mild dementia	Cognitive function: TICS and CDR Depression: GDS-15 Apathy: Not measured	IG: 3.27 (2.84) CG: 3.17 (2.72)		IG: Cognitive rehabilitation techniques with multi-family group (CA) CG: Standard care	Medication management ability assessment, bill paying subtest from the executive function performance, activities of daily living-prevention instrument, coping self-efficacy scale	GDS score of both groups of participants with MCI did not show a significant difference (*p* = 0.07).	Ineffective
Treusch et al. (2015)	IG: *n* = 67 CG: *n* = 50	Nursing home	Dementia patients with apathy	Severe	Cognitive function: MMSE Depression: DMAS Apathy: AES, NPI	IG: 17.41 (14.19) CG: 15.48 (12.10)	AES IG: 50.01 (11.17) CG: 47.38 (8.87)	IG: Occupational therapy in the form of a “biographically orientated mobilization” (SA) CG: No special intervention	Apathy	AES score of IG was maintained at pre-post intervention, but CG was worsened (*p* = 0.01). DMAS and NPI were not used as the outcome of the intervention.	Effective
Valentí Soler et al. (2015)	Phase 1 Nursing home IG1: *n* = 30 IG2: *n* = 33 CG: *n* = 38 Day care center IG: *n* = 20 Phase 2 Nursing home: IG 1: *n* = 36 IG2: *n* = 42 CG: *n* = 32 Day care center: IG2: *n* = 17	Nursing home and day care center	People with dementia	Mild to severe	Cognitive function: MMSE Depression: Not measured Apathy: NPI, APADEM-NH, AI		Phase 1 Nursing home APADEM-NH IG1: 45.06 (20.69) IG2: 48.40 (19.12) CG: 43.21 (21.80) NPI IG1: 8.85 (2.55) IG2: 9.26 (2.28) CG: 8.73 (2.54)	Phase 1 Nursing home IG1: NAO (Humanoid) (SA) IG2: PARO (Animal) (SA) CG: Conventional therapy Day care center IG: NAO (SA) Phase 2 Nursing home IG1: Dog (real animal) (SA) IG2: PARO (SA) CG: Conventional therapy Day care center IG2: PARO (SA)	Apathy	APADEM-NH total scores in IG1 (*p* = 0.030) and IG2 (*p* = 0.049) and NPI apathy subscale score in IG1 (*p* = 0.047) showed a significant decrease at Phase 1 in nursing home.	Effective
Van Bogaert et al. (2016)	IG: *n* = 29 CG: *n* = 31	Nursing home	Residents with dementia	Mild to moderate	Cognitive function: MMSE Depression: CSDD Apathy: Not measured	IG: 5 (2 – 8) CG: 3 (1 – 5) Note. Median (Inter-Quartile Range)		IG: Standardized individual reminiscence intervention based on SolCos model (EA) CG: usual care	Depressive symptoms	Delta score of CSDD showed a significant difference between IG and CG (Δ = −4, *p* < 0.05), but linear regression analysis did not show the effect of the intervention (*b* = −2.37, 95% CI [−4.81, 0.06], *p* = 0.056).	Ineffective
Wang et al. (2010)	IG: *n* = 16 CG: *n* = 13	Outpatient clinic at hospital	Patients with CVD	Mild	Cognitive function: MMSE Depression: GHQ Apathy: Not measured	IG: 2.13 (2.17) CG 1.33 (1.72)		IG: Tai Chi exercise (SA) CG: Rehabilitation (SA)	P300, GHQ, sleep quality	GHQ severe depression subscale score was significantly improved in the IG (time*group interaction, *F* = 6.143, *p* = 0.02).	Effective

AD, Alzheimer’s Disease; AES, Apathy Evaluation Scale; AI, Apathy Inventory; APADEM-NH, the Apathy Scale for Institutionalized Patients with Dementia-Nursing Home version; CDR, Clinical Dementia Rating; CA, Cognition-oriented Approaches; CG, Control Group; CSDD, Cornell Scale for Depression with Dementia; CST, Cognitive Stimulation Therapy; CVD, Cerebral Vascular Disorder; DMAS, Dementia Mood Assessment Scale; EA, Emotion-oriented Approaches; GDS, Geriatric Depression Scale; GHQ, General Health Questionnaire; IADL, Instrumental Activities of Daily Living; IG, Intervention Group; MCI, Mild Cognitive Impairment; MoCA, Montreal Cognitive Assessment; MMSE, Mini-Mental State Examination; NPI, Neuropsychiatric Inventory; OA, Other Approaches; QOL, Quality of Life; RMBPC, Revised Memory and Behavior Problem Checklist; SA, Stimulation-oriented Approaches; SD, Standard Deviation; SE, Standard error; and TICS, Telephone Interview of Cognitive Status.

### Characteristics of Participants

Most of the participants were recruited from care facilities (e.g., nursing homes; Hsieh et al., 2010; Brooker et al., 2011; Van Bogaert et al., 2013; Alves et al., 2014; Treusch et al., 2015; Valentí Soler et al., 2015; Olsen et al., 2016; Huang et al., 2019; Pérez-ros et al., 2019), their community (Schmitter-Edgecombe and Dyck, 2014; Larouche et al., 2019; Lai et al., 2020), and hospitals (Wang et al., 2010; Hattori et al., 2011; Bergamaschi et al., 2013; Pongan et al., 2017; Huang et al., 2019; Gomez-Soria et al., 2020; Reverté-villarroya et al., 2020). Participants recruited from the medical institute were diagnosed with the subtypes of dementia. The MMSE, MoCA, and CDR were used to evaluate the cognitive status of the participants. Participants with MCI or mild-to-moderate dementia were the main target of the intervention in these studies, with only few studies recruiting participants with severe dementia (Treusch et al., 2015; Valentí Soler et al., 2015; Olsen et al., 2016). Fifteen studies evaluated depression, two evaluated apathy, and two evaluated the effect of the intervention. Overall, scores on the depression and apathy scales were low, except for some studies that included unclear versions of the scale as well as unclear cut-off points (Hsieh et al., 2010; Brooker et al., 2011; Hattori et al., 2011; Schmitter-Edgecombe and Dyck, 2014; Fernández-Calvo et al., 2015; Valentí Soler et al., 2015; Olsen et al., 2016; Pongan et al., 2017; Larouche et al., 2019; Lin et al., 2019; Pérez-ros et al., 2019; Gomez-Soria et al., 2020; Reverté-villarroya et al., 2020).

### Assessment Tools to Evaluate Depression and Apathy

The Geriatric Depression Scale (GDS; Hsieh et al., 2010; Brooker et al., 2011; Hattori et al., 2011; Alves et al., 2014; Schmitter-Edgecombe and Dyck, 2014; Pongan et al., 2017; Huang et al., 2019; Larouche et al., 2019; Pérez-ros et al., 2019; Gomez-Soria et al., 2020; Reverté-villarroya et al., 2020) and the Cornell Scale for Depression with Dementia (CSDD; Bergamaschi et al., 2013; Fernández-Calvo et al., 2015; Olsen et al., 2016; Van Bogaert et al., 2016; Lin et al., 2019; Pérez-ros et al., 2019) were most frequently used to assess the depressive symptoms of the participants. The Starkstein Apathy Scale (SAS; Hattori et al., 2011), Apathy Inventory (Valentí Soler et al., 2015), and Apathy Evaluation Scale (AES; Hsieh et al., 2010; Treusch et al., 2015) were used to evaluate apathy. The Neuropsychiatric Inventory (NPI; Hsieh et al., 2010; Treusch et al., 2015; Valentí Soler et al., 2015; Reverté-villarroya et al., 2020) was used to assess both symptoms.

### Effect of Non-pharmacological Interventions

Of the 20 extracted studies, only seven (two on apathy, four on depression, and one on both) clearly set depression and apathy as the primary outcome (Hsieh et al., 2010; Van Bogaert et al., 2013; Treusch et al., 2015; Valentí Soler et al., 2015; Olsen et al., 2016; Larouche et al., 2019; Pérez-ros et al., 2019). The studies that did not set either of these symptoms as the primary outcome aimed to investigate the effect of the intervention program on cognitive function, various BPSD not limited to depression or apathy, physical function, etc.

Of the 18 studies that evaluated depression, 11 (61.1%) were judged to be effective interventions. These included animal-assisted activities (Olsen et al., 2016), preference for listening to music (Pérez-ros et al., 2019), Tai Chi exercise (Wang et al., 2010; Huang et al., 2019), a painting intervention (Pongan et al., 2017), a multi-intervention program (Brooker et al., 2011; Fernández-Calvo et al., 2015; Lai et al., 2020), reminiscence group therapy (Hsieh et al., 2010), mindfulness- and psychoeducation-based interventions (Larouche et al., 2019), and creative expression therapy (Lin et al., 2019). In contrast, non-effective interventions included standard cognitive stimulation therapy (Alves et al., 2014), cognitive training (Bergamaschi et al., 2013; Gomez-Soria et al., 2020), educational nursing intervention in the family (Reverté-villarroya et al., 2020), cognitive rehabilitation with a multi-family group (Schmitter-Edgecombe and Dyck, 2014), individual reminiscence therapy (Van Bogaert et al., 2016), and art therapy (Hattori et al., 2011).

All four studies that evaluated apathy revealed that the interventions were effective, including biographically oriented mobilization (Treusch et al., 2015), art therapy (Hattori et al., 2011), reminiscence group therapy (Hsieh et al., 2010), and robot-assisted intervention (Valentí Soler et al., 2015).

## Discussion

This study explored effective non-pharmacological interventions on apathy and depression among individuals with MCI and dementia. Interventions judged as “effective” included emotion- and stimulation-oriented approaches. Although apathy and depression can coexist, studies that targeted both symptoms were surprisingly few. Although various approaches were employed to improve physical and psychological functions, many interventions did not set apathy and depression as the primary outcomes. The lack of evidence regarding the effect of non-pharmacological interventions on depression and apathy may be attributed to this.

Almost all of the effective interventions for apathy and depression employed the emotion-oriented approach, which included reminiscence therapy; the stimulation-oriented approach, which included art therapy (American Psychiatric Association, 2007); or a combination of these approaches. Individuals with depression but without apathy, when compared to those with apathy, exhibit relatively higher levels of depression and lethargy (Batail et al., 2018). This suggests that people with depression but without apathy are distinct from those with depression and apathy in terms of their emotional symptoms. Apathy is conceptualized as the loss of motivation, which includes cognitive, emotional, and auto-activating aspects (Levy and Dubois, 2006). According to the proposed criteria for apathy, loss of or diminished goal-directed behavior can be divided into two sub-domains: self-initiated behavior and environment-stimulated behavior (Robert et al., 2009). Emotion- or stimulation-oriented approaches provide environmental stimulation even if patients cannot self-initiate behavior, which may be appropriate to directly encourage the emotional or behavioral aspects of depression and apathy. However, because most studies did not set depression or apathy as the primary outcome, the effect of the non-pharmacological interventions may be distorted. Therapists conducting non-pharmacological interventions should expend effort in selecting the most suitable intervention for specific symptoms.

Participants in some studies were not diagnosed with subtypes of dementia, which may be attributed to the fact that these interventions were conducted at long-term care facilities or the community, not at a medical institution. Previous studies have indicated that older adults living in long-term care facilities or the community often do not receive a formal diagnosis of dementia even if they show clear cognitive impairment (Bartfay et al., 2013; Lang et al., 2017). However, the features of BPSD depend on the difference in the dementia subtypes (Kazui et al., 2016). For instance, people having dementia with Lewy bodies at the very mild stage showed more severe depressive symptoms and apathy than those with Alzheimer’s disease (Hashimoto et al., 2015), suggesting that the effect of the non-pharmacological intervention may also differ depending on the dementia subtypes. Although all studies that included a diagnosis of the dementia subtypes in this review addressed Alzheimer’s disease, except for one that targeted cerebral vascular disorder, studies that consider the subtypes of dementia are better suited to assess the effectiveness of interventions for apathy and depression.

The severity of cognitive impairment or apathy and depression may also influence the effect of the intervention. Many extracted studies that targeted severe dementia were not included, as the effect of the interventions was unclear. Few studies targeted severe dementia, possibly due to a publication bias—interventions for severe dementia are likely to be ineffective. Some studies targeted participants with MCI. For instance, although our findings suggested that the mindfulness-based approach is effective for treating apathy and depression, participants of the study included individuals with MCI and not dementia (Larouche et al., 2019). A systematic review suggested that the effectiveness of mindfulness-based interventions is unclear in moderate or severe dementia (Chan et al., 2020). Likewise, participants in many studies obtained a low score in the apathy and depression scale at baseline, which may also have influenced the effect of the intervention. Since the prevalence of BPSD is also influenced by the severity of cognitive impairment (Zhang et al., 2012; Leung et al., 2021), further studies should consider the influence of this severity on apathy and depression while evaluating non-pharmacological approaches.

The NPI is often used to assess BPSD because it ensures concise evaluation of the frequency and severity of various aspects of BPSD. Although some studies in this scoping review also employed the NPI to assess apathy and depression, it did not focus on specific characteristics of apathy and depression. Unlike the NPI, the GDS, CSDD, and AES can provide a deeper assessment of apathy and depression because these scales comprise multiple sub-factors (Faerden et al., 2008; Hsieh et al., 2012; Kim et al., 2013; Barca et al., 2015). Since apathy and depression coexist (Levy and Dubois, 2006), analysis based on the sub-factors may be helpful to differentiate between their symptoms. Therefore, the assessment scales that cover the various factors of apathy and depression are acceptable for evaluating the effect of interventions for depression and apathy.

There are several limitations to the present review. First, this review did not determine the quality of each study, which may have influenced the results. Further, limitations in the sample size or the statistical techniques could have distorted the effects of the intervention, thus influencing its clinical significance. Further information regarding the effect size is required for an accurate interpretation of the results. Second, although some interventions were compared with usual care, most studies did not describe usual care in detail. Daily care involves various activities for patients with dementia. Thus, it would be necessary to describe usual care in detail when it is used for the control group. Third, although the present study employed the framework of American Psychiatric Association (2007), Work Group on Alzheimer’s Disease and Other Dementias, the extent to which the care was personalized or person-centered is also an important factor that might influence the non-pharmacological intervention (Chenoweth et al., 2009; Lu et al., 2021). Future research would be required to address these factors while examining the effects of the interventions. Despite these limitations, this review provides helpful findings for practitioners to select non-pharmacological interventions for individuals with depression and apathy with dementia.

Assessment and early interventions for people with dementia who exhibit apathy and depression are important in improving their quality of life. As a clinical implication, the findings suggest that emotion- or stimulation-oriented approaches may be useful, particularly in people with MCI and mild-to-moderate dementia. Our findings also suggest that future studies, especially systematic reviews and meta-analyses, should be focused on setting apathy and depression as the primary outcomes of non-pharmacological interventions.

## Author Contributions

HO researched the literature and wrote the draft of the paper. RK, SK, KS, KO, and KI provided insightful comments to analyze the extracted studies and improve the draft of the paper. All authors were involved in writing the manuscript and approve of its publication.

## Funding

This study was supported by the Health Labour Sciences Research Grant (grant number: 20GB1002).

## Conflict of Interest

The authors declare that the research was conducted in the absence of any commercial or financial relationships that could be construed as a potential conflict of interest.

## Publisher’s Note

All claims expressed in this article are solely those of the authors and do not necessarily represent those of their affiliated organizations, or those of the publisher, the editors and the reviewers. Any product that may be evaluated in this article, or claim that may be made by its manufacturer, is not guaranteed or endorsed by the publisher.
